# Clinical and genetic features of maturity-onset diabetes of the young in pediatric patients: a 12-year monocentric experience

**DOI:** 10.1186/s13098-021-00716-6

**Published:** 2021-09-08

**Authors:** Stefano Passanisi, Giuseppina Salzano, Bruno Bombaci, Fortunato Lombardo

**Affiliations:** Department of Human Pathology in Adult and Developmental Age “Gaetano Barresi”, University of Messina, Via Consolare Valeria 1, 98124 Messina, Italy

**Keywords:** C-peptide, Diagnosis, Genetic testing, Glucokinase, MODY, Sulfonylureas

## Abstract

**Background:**

A retrospective observational study was conducted to assess the prevalence of maturity onset diabetes of the young (MODY) in a large paediatric population of Southern Italy newly diagnosed with diabetes. Clinical and genetic features of the identified MODY patients were also described.

**Methods:**

Genetic testing was performed in children and adolescents newly diagnosed with diabetes who presented autoantibody negativity and fasting C-peptide levels ≥ 0.8 ng/mL. Patients with a low insulin daily dose and optimal glycaemic control after two years from diabetes onset were also investigated for monogenic diabetes, regardless of their autoimmunity status and/or C-peptide levels.

**Results:**

A prevalence of 6.5% of MODY was found. In particular, glucokinase-MODY was the most common type of MODY. The mean age at diagnosis was 9.1 years. Clinical presentation and biochemical data were heterogeneous also among patients belonging to the same MODY group.

**Conclusions:**

We found a relatively high prevalence of MODY among paediatric patients with a new diagnosis of diabetes in comparison to literature data. Our findings highlight that a more detailed clinical evaluation along with easier and less expensive approachability to genetic testing may allow diagnosing an increasing number of MODY cases. A correct, prompt diagnosis is crucial to choose the most appropriate treatment and offer adequate genetic counselling.

## Introduction

Maturity onset diabetes of the young (MODY) is a rare condition characterized by autosomal dominant inheritance which is usually diagnosed before 25 years of age [1]. According to the latest clinical practice recommendations of the American Diabetes Association, MODY belongs to the monogenic diabetes syndromes [2]. It is frequently characterized by onset of hyperglycaemia caused by impairment of insulin secretion, with minimal or no defects in insulin action [3]. To date, at least 14 different genes have been reported to cause diabetes with a MODY-like phenotype [4]. Each MODY subtype is associated to different patterns (e.g. age of onset, clinical features, type of treatment) [5]. Although the traditional criteria for MODY include a family history of diabetes, sporadic de novo mutations in a number of causative genes have been reported [6–8].

MODY accounts for 1–5% of all cases of diabetes and 1–6% of paediatric diabetes cases [9–13]. Mutations in the hepatocyte nuclear factor 1-alpha (*HNF1A*), glucokinase (*GCK*), hepatocyte nuclear factor 4-alpha (*HNF4A*), and hepatocyte nuclear factor 1-beta (*HNF1B*) genes are the most frequently identified aetiologies of MODY. Other MODY genes include: *PDX1*, *HNF1B*, *NEUROD1*, *KLF11*, *CEL*, *PAX4*, *INS*, *BLK*, *ABCC8*, and *KCNJ11*.

GCK-MODY, known as MODY 2, is the commonest subtype of monogenic diabetes in paediatric age, especially among Caucasian populations [11]. The GCK-MODY phenotype is characterized by mild fasting hyperglycaemia since birth, typically in the range of 5.5–8.0 mmol/l, which tends to remain stable or to increase only marginally with age [14]. The *GCK* mutational spectrum includes over 700 mutations distributed throughout the gene [15]. Patients with GCK-MODY do not usually require any pharmacological treatment [4]. HNF1A-MODY, also known as MODY-3, is more frequent in the United Kingdom and the Netherlands [16, 17]. Hyperglycaemia is the result of progressive beta-cell function deterioration over time in the absence of clinical signs of insulin resistance. Rarely, this type of MODY becomes apparent with mild-to-moderate ketoacidosis. Glycosuria is often present and is related to a low renal threshold, typically < 10 mmol/l [18]. Thus far, more than 400 causative mutations have been reported, and they are mainly found in exon 2 and exon 4 of the gene [19, 20]. HNF4A-MODY, also called MODY 1, is caused by heterozygous mutations in the *HNF4A* gene, resulting in a progressive β-cellular dysfunction [21]. HNFA4-MODY differs from HNF1A-MODY due to absence of glycosuria, older age at diagnosis, presence of reduced levels of apolipoproteins (AII and CIII), lipoprotein A and triglycerides and, above all, presence of macrosomia (50–60% of cases) and transient neonatal hyperinsulinemic hypoglycemia responsive to treatment with diazoxide [14]. More than 100 *HNF1A* mutations have been described [22]. Patients with HNF4A-MODY and HNF1A-MODY are generally sensitive to low-dose sulfonylurea [5].

Aim of this study was to assess the prevalence of MODY among a large paediatric population of Southern Italy newly diagnosed with diabetes. Secondary outcome of the study was to describe the main clinical and genetic features of the identified MODY patients.

## Material and methods

A retrospective analysis was conducted to select all patients who were admitted at our Paediatric Centre of Diabetes between January 2009 and February 2021 with a new diagnosis of diabetes. The study was conducted in accordance with the Helsinki Declaration, good clinical practice and all applicable laws and regulations. The study protocol was approved by the (local) Ethics Committee of the Medical University of Messina. The diagnosis of diabetes was made according to the current criteria of the ISPAD Clinical Practice Consensus Guidelines: classic symptoms of diabetes or hyperglycaemic crisis, with plasma glucose concentration ≥ 11.1 mmol/L (200 mg/dL) or fasting plasma glucose ≥ 7.0 mmol/L (≥ 126 mg/dL) or two hour postload glucose ≥ 11.1 mmol/L (≥ 200 mg/dL) during an oral glucose tolerance test (OGTT) or glycated haemoglobin (HbA1c) > 6.5% (48 mmol/mol) [4]. Data analysed included patient demographic and clinical variables.

Diabetes-specific islet cell autoantibodies (ICA), glutamic acid decarboxylase 65 autoantibodies (GADA), tyrosine phosphatase-like insulinoma antigen 2 (IA2), insulin (IAA), and β-cell-specific zinc transporter 8 autoantibodies (ZnT8) were measured. The presence of ICA was determined by immunofluorescence assay, while GADA, IA2, IAA, and ZnT8 were evaluated with enzyme linked immunosorbent assay. The identification of predisposing human leukocyte antigen (HLA) alleles (i.e. HLA-DRB1, HLA-DQA1, and HLA-DQB1) was also performed in every patient. In cases with the suspicion of monogenic diabetes, genetic tests were performed. In accordance with previous studies [13], selection criteria for genetic investigations included diabetes autoantibody negativity and fasting C-peptide levels ≥ 0.8 ng/mL at time of diagnosis. However, some patients with a low insulin daily dose (daily insulin dose < 0.5 IU/kg) and optimal glycaemic control (HbA1c < 6.5% or 48 mmol/mol) after two years from diabetes onset were also investigated for monogenic diabetes, regardless of their autoimmunity and/or C-peptide levels.

### Genetic testing

Genetic testing for monogenic diabetes was performed in two different laboratories located in Reggio Calabria and San Giovanni Rotondo, Italy. Analysis of monogenic diabetes causing genes was performed using the “HaloPlex HS Target Enrichment System” for sample preparation, which was thereafter sequenced on NextSeq 500 through 2 × 150 bp paired-end sequencing. The generated sequencing data was mapped to the human genome reference hg19. Thirty genes were analysed: *ABCC8*, *APPL1*, *BLK*, *CEL*, *GATA4*, *GATA6*, *GCK*, *HNF1A*, *HNF1B*, *HNF4A*, *INS*, *PDX1*, *KCNJ11*, *KLF11*, *LMNA*, *NEUROD1*, *PAX4*, *PPARG*, *RFX6*, *TRMT10A*, *WFS1*, *ZFP57*, *EIF2AK3*, *FOXP3*, *GLIS3*, *IER3IP1*, *NEUROG3*, *PTF1A*, *SLC19A2*, *SLC2A2*. Raw data obtained from the genetic investigations were evaluated according to American College of Medical Genetics and Genomics guidelines. Confirmation studies were performed for variants that were considered to be pathogenic, or likely pathogenic, using Sanger sequencing.

## Results

During the study period, a total of 565 children and adolescents were newly diagnosed with diabetes. Genetic tests for monogenic diabetes were performed in 68 patients. Of these, 59 (86.8%) were negative for diabetes-specific autoantibodies, 46 (67.6%) had fasting C-peptide level ≥ 0.8 ng/mL, 63 (92.6%) were not on insulin therapy or required insulin daily dose < 0.5 IU/kg along with an optimal glycaemic control (HbA1c < 6.5% or 48 mmol/mol).Thirty-seven patients (6.5%) were diagnosed as MODY. In particular, 2 had a diagnosis of HNF4A-MODY (5.4%), 30 were diagnosed as GCK-MODY (81%) and the remaining 5 were HNF1A-MODY (13.5%). Mean age at diagnosis of MODY was 9.1 years (ranging from 0 to 16 years). Genotypic and phenotypic features of MODY patients are summarized in Tables 1 and 2. Other diagnosis included type 1 diabetes (T1D) (91.1%), type 2 diabetes (T2D) diagnosed according to the American Diabetes Association criteria in 10 patients (1.9%) [2], Wolfram syndrome (0.3%) and mitochondrial diabetes (0.2%).

**Table 1:** Mutations and genotype of study MODY patients

N	Diagnosis	Exon	Type of mutation	Nucleotide change	Protein effect	Known/Novel
1	HFN4A-MODY	3	Frameshift	c.364_365insG	p.Ala123fs	Novel
2	HFN4A-MODY	3	Frameshift	c.364_365insG	p.Ala123fs	Novel
3	GCK-MODY	10	Frameshift	c.440_450del	p.His147fs	Known
4	GCK-MODY	2	Missense	c.106C > T	p.Arg36Trp	Known
5	GCK-MODY	7	Frameshift	41756insG	Unknown	Novel
6	GCK-MODY	7	Frameshift	41756insG	Unknown	Novel
7	GCK-MODY	5	Nonsense	c.559C > T	p.Arg187Trp	Known
8	GCK-MODY	6	Missense	c.674G > A	p.Arg225His	Novel
9	GCK-MODY	8	Missense	c.955G > A	p.Gly318Arg	Known
10	GCK-MODY	8	Missense	c.955G > A	p.Gly318Arg	Known
11	GCK-MODY	6	Missense	c.682G > C	p.Thr228Ala	Known
12	GCK-MODY	6	Missense	c.682G > C	p.Thr228Ala	Known
13	GCK-MODY	6	Missense	c.682G > C	p.Thr228Ala	Known
14	GCK-MODY	7	Missense	c.781G > A	p.Gly261Arg	Known
15	GCK-MODY	8	Missense	c.955G > A	p.Gly318Arg	Known
16	GCK-MODY	5	Nonsense	c.556C > T	p.Arg186Ter	Known
17	GCK-MODY	5	Nonsense	c.556C > T	p.Arg186Ter	Known
18	GCK-MODY	Intron 9	Splice site	IVS9-2A > G	Unknown	Novel
19	GCK-MODY	Intron 9	Splice site	IVS9-2A > G	Unknown	Novel
20	GCK-MODY	5	Missense	c.538A > G	p.Asn180Asp	Known
21	GCK-MODY	5	Missense	c.544G > A	p.Val182Met	Known
22	GCK-MODY	5	Frameshift	Deletion of 21 nucleotides	Unknown	Known
23	GCK-MODY	2	Missense	c.113A > C	p.Gln38Pro	Known
24	GCK-MODY	Intron 1	Splice site	IVS1 + 1G > T	Unknown	Novel
25	GCK-MODY	Intron 1	Splice site	IVS1 + 1G > T	Unknown	Novel
26	GCK-MODY	9	Frameshift	Deletions of 21 nucleotids	Unknown	Known
27	GCK-MODY	5	Nonsense	c.3989-9G > A	Unknown	Novel
28	GCK-MODY	7	Missense	c.784G > A	p.Gly262Arg	Known
29	GCK-MODY	10	Missense	c.1261A > G	p.Ser131Pro	Known
30	GCK-MODY	7	Missense	c.796G > A	p.Glu265Lys	Known
31	GCK-MODY	7	Missense	c.796G > A	p.Glu265Lys	Known
32	GCK-MODY	2	Missense	c.116A > C	p.Gln38Pro	Known
33	HFN1A-MODY	1	Missense	c.229G > A	p.Asp77Asn	Novel
34	HFN1A-MODY	1	Missense	c.229G > A	p.Asp77Asn	Novel
35	HFN1A-MODY	7	Missense	c.1340C > T	p.Pro447Leu	Known
36	HFN1A-MODY	3	Missense	c.709A > G	p.Asn237Asp	Known
37	HFN1A-MODY	4	Missense	c.815G > A	p.Arg272His	Known

**Table 2:** Clinical characteristics of study GCK-MODY and HFN1A-MODY patients

	GCK-MODY	HNF1A-MODY
Number of patients	30 (81%)	5 (13.5%)
Age at diagnosis (yrs)	8.5 ± 4.5	9.9 ± 5.6
BMI at diagnosis (Z-score)	− 0.2 ± 1.1	0.6 ± 1.16
HbA1c at onset (%)	6.5 ± 0.5	7.3 ± 2.2
HbA1c at onset (mmol/mol)	48 ± 6	56 ± 24
OGTT: 0ˈ glucose (mmol/l)	6.26 ± 0.93	7.02 ± 0.98
OGTT: 120ˈ glucose (mmol/l)	9.22 ± 1.89	14.15 ± 4.83
C-peptide at onset (ng/dl)	0.8 ± 0.5	1.8 ± 1.0
Weight at birth (kg)	2.95 ± 0.6	2.90 ± 0.5
HLA		
DR3 and/or DR4	10 (33.3%)	1 (20%)
Autoantibodies		
ICA	1	0
IA2	1	0
GADA	2	0
Treatment		
Diet only	29	1
Insulin	1	2
OHA	0	2

*BMI* Body Mass Index, *GADA* glutamic acid decarboxylase 65 autoantibodies, *HbA1c* glycated haemoglobin, *HLA* human leukocyte antigens, *IA2* tyrosine phosphatase-like insulinoma antigen 2 autoantibodies, *ICA* islet cell autoantibodies, *OGTT* oral glucose tolerance test, *OHA* oral hypoglycemic agents

### HFN4A-MODY

Two siblings (a male and a female) had a diagnosis of *HFN4A*-MODY at the age of 16 and 12, respectively. Diagnostic investigations started after discovering an occasional hyperglycaemia in one of the two siblings. At that time, her HbA1c was 6.1% (43 mmol/mol) and an OGTT confirmed the diagnosis of diabetes (2-h postload blood glucose 12.1 mmol/l). Levels of the diabetes-specific autoantibodies were all negative. HLA was not predisposing to T1D. On the suspicion of monogenic diabetes, genetic testing was performed. Genetic analysis revealed a frameshift mutation (*HNF4A* c.364_365insG) in exon 3 of the *HNF4A* gene. Thus, genetic investigations were extended to her families, revealing the same mutation in her brother, who was asymptomatic. Both siblings had normal liver function without morpho-structural alterations. Treatment with gliclazide was started at low daily dose. Thus far, after a 5-year-follow-up, both patients have optimal glycaemic control—last year HbA1c value < 6% (42 mmol/mol)—thus, oral hypoglycaemic agent withdrawal has been tried.

### GCK-MODY

A total of 30 children were identified as GCK-MODY during the study period. Of these, 13 were male. Mean BMI Z-score at start of follow-up was − 0.2 ± 1.1. Genetic analysis leading to diagnosis of monogenic diabetes was performed at the age of 8.5 ± 4.5 years. Mean HbA1c at diagnosis was 6.5 ± 0.5% (48 ± 6 mmol/mol), while basal C-peptide level was 0.8 ± 0.5 ng/ml. Oral glucose tolerance tests of these patients showed a fasting blood glucose of 6.26 ± 0.93 mmol/l with a postload blood glucose after 120 min of 9.22 ± 1.89 mmol/l. All patients were investigated for diabetes-specific autoantibodies and the presence of T1D predisposing HLA: 2 patients were positive for GADA and one of these also had ICA and IA2; 10 patients showed a predisposing HLA. Mean weight at birth of the components of this group was 2.95 ± 0.6 kg. Of note, one patient diagnosed with GCK-MODY at the age of 6 years was also affected by cystic fibrosis [23]. An infant with a known family history of GCK-MODY (mother and her elder brother were affected) received a prenatal diagnosis after amniocentesis was performed. Almost all these patients are treated with a balanced diet regimen and regular physical activities without any pharmacological treatment. Only one patient started subcutaneous insulin therapy due to a brittle glycaemic control after 6 years from diagnosis. Gene sequencing revealed a missense mutation in the majority of cases. Frameshift, nonsense and splice site mutations were also identified. Sites of mutation were in exons 5, 6 and 7 in more than half the cases.

### HFN1A-MODY

Five patients with *HFN1A* gene mutation were found in our study population. Their clinical features are summarized in Table 3. Two of these are siblings. Age at time of diagnosis ranged from 2 to 17 years. Diabetes onset in a 10-year-old girl was characterized by the occurrence of polyuria, polydipsia, and headache. Her glycaemia was 21.4 mmol/l, HbA1c 10.7% (94 mmol/mol). Ketonemia and ketonuria were negative. No family history of diabetes was present. Genetic investigations revealed a de novo missense mutation in the exon 3 of *HFN1A* gene (*HFN1A*-c.709A > G) [8]. Other patterns of clinical presentation varied from hypoglycaemic episodes to hyperglycaemia. One out of 5 patients was obese at time of diagnosis (Z-score BMI 1.7). Overall, BMI Z-score ranged from − 1.10 to 1.7. HbA1c determination at onset was heterogeneous: in addition to the patient previously described, 2 patients showed a level above the range—7.5% (58 mmol/mol) and 7.6% (60 mmol/mol)—while the remaining 2 were in the normal range: 5.1% (32 mmol/mol) and 5.4% (36 mmol/mol). Mean fasting and two hour postload glucose levels were respectively 7.02 ± 0.98 mmol/l and 14.15 ± 4.83 mmol/l. Basal C-peptide was in the normal range except for one patient who showed a value of 0.30 ng/dl. None of the patients were positive for T1D-specific autoantibodies. One case presented T1D predisposing heterozygous allele HLA DR4. Two of our HFN1A-MODY patients (siblings) currently practice subcutaneous insulin therapy due to failure with sulfonylurea treatment, while another two are on therapy with gliclazide. The remaining one patient with a recent diagnosis of HFN1A-MODY is still on lifestyle treatment because of satisfactory glycaemic control. Gene sequencing detected a missense mutation of the *HFN1A* gene affecting exons 1, 3, 4 and 7 in every patient.

**Table 3:** Clinical characteristics of study HFN1A-MODY patients

	Patient 1	Patient 2	Patient 3	Patient 4	Patient 5
Age at diagnosis (ys)	2.5	7.2	17.8	11.3	10.7
BMI at diagnosis (Z-score)	0.5	0.2	1.4	-1.1	1.9
HbA1c at onset (%)	5.1	5.5	7.4	10.7	7.3
HbA1c at onset (mmol/mol)	32	37	57	93	56
OGTT: 0ˈ glucose (mmol/l)	N/A	5.89	7.67	N/A	7.50
OGTT: 120ˈ glucose (mmol/l)	N/A	8.94	15.0	N/A	18.50
C-peptide at onset (ng/dl)	0.30	2.66	1.38	2.84	2.00
Weight at birth (kg)	2.720	3.150	2.440	3.680	2.500
HLA					
DR3 and/or DR4	No	Yes	No	No	No
Autoantibodies	No	No	No	No	No
Treatment	Insulin	Insulin	Diet	Gliclazide	Gliclazide

*BMI* Body Mass Index, *HbA1c* glycated haemoglobin, *HLA* human leukocyte antigens, *OGTT* oral glucose tolerance test

## Discussion

Over the past two decades, improved knowledge and awareness of monogenic diabetes, along with the development of increasingly more accurate and worldwide available molecular diagnostic techniques, have led to an increased estimate of the prevalence of MODY. The identification of genetic diagnosis is extremely important when choosing the most proper treatment plan. It is well established that patients affected by GCK-MODY do not require any pharmacological treatment [15], while patients with HNF1A or HNF4A-MODY have a good response to sulfonylureas in term of glycaemic control and prevention of micro- and macrovascular complications [24]. A recent cost-effectiveness model suggested that a screening approach would be cost-saving, and the savings would increase for every additional family member who could be identified [25]. Therefore, given the clinical and economic benefits of a timely diagnosis, it is critical to avoid potential delays between diabetes diagnosis and molecular confirmation of MODY, particularly in paediatric patients [26].

Our 12-year-long experience identified a prevalence of MODY of 6.5% among a population of children and adolescents newly diagnosed with diabetes. This finding is consistent with a previous Italian multicentre study, which found a prevalence of 5.5% [27]. Conversely, other studies on paediatric diabetic populations revealed lower rates of prevalence ranging from 0.65 to 2.5% [13, 28–32]. The gap between these prevalence rates may be explained by different selection criteria adopted for patients undergoing genetic tests for MODY. Several studies on paediatric diabetic populations have systematically used diabetes-specific autoantibody negativity in combination with other biochemical parameters (i.e. basal C-peptide value and urinary C-peptide creatinine ratio) as screening methods to identify eligible patients for genetic tests for MODY [13, 32]. In an observational U.S. multicentre study, only patients with absence of diabetes-specific autoimmunity and C-peptide levels within the normal range were genetically investigated, resulting in a prevalence of MODY of 1.2% among children and adolescents with diabetes [13]. Similarly, Shepherd et al. [32] screened 808 UK children with diabetes. Only patients who had negative T1D autoantibodies and normal urinary C-peptide creatinine ratio underwent genetic tests, resulting in 20 patients diagnosed with MODY out of 82 selected children. Conversely, Johnson et al. found a prevalence of 2.1% in 821 Australian paediatric patients with diabetes screened for MODY regardless of their clinical features [30].

In our centre, genetic investigations for monogenic diabetes were performed in presence of a clinical suspicion of non-T1D, without any strict criteria. We can speculate that serum C-peptide value alone is too stringent as it is well-known to be correlated with patients’ age and BMI [33]. Basal serum C-peptide was low in many of our patients affected by MODY, especially in the GCK-MODY subgroup. Furthermore, we found that diabetes-specific autoantibodies were positive in 2 patients diagnosed with MODY. GADA were present in both patients. GADA, especially alone, have a low predictive value for T1D when compared to other autoantibodies [34]. Existing evidence has shown a comparable incidence of positivity to autoantibodies between patients with MODY and the general population, of approximately 1% [35]. Finally, we can affirm that if we had excluded patients from gene sequencing on the basis of these strict criteria, we would have missed 13 diagnoses.

Interestingly, we found a relatively high number of patients belonging to the MODY group who presented HLA haplotype predisposing to T1D. Data from other studies show that the presence of HLA alleles DR3 and DR4 is not rare among MODY patients, ranging from 20 to 35.1% [31, 36]. The presence of HLA alleles DR3 or DR4 has been previously investigated as a discriminating factor between T1D and MODY [37].

Regarding the frequency among MODY forms, most of our MODY patients have *GCK* gene mutations, confirming previous evidence among Caucasian populations [27, 28, 30, 31, 38]. Discordance with other studies showing a higher prevalence of HNF1A-MODY [12, 13, 29] could be related to differences between national healthcare systems [26]. The Italian health service provides universal coverage to residents and public healthcare is largely free of charge. GCK-MODY is characterized by mild clinical signs that could be overlooked resulting in failure to diagnose unless the patient accesses tertiary diabetes outpatient services. This could explain its higher prevalence in countries providing easier access to tertiary care, in comparison to countries with other forms of public health, such as insurance-based ones.

In our study, mean HbA1c at diagnosis in GCK-MODY patients was lower than in HNF1A-MODY, confirming the results of another study [39], in which the assessment of HbA1c in combination with fasting glucose was proposed in the differential diagnosis between these two subtypes in subjects with suspect monogenic diabetes. In particular, those authors suggested considering HNF1A-MODY as the first hypothesis in patients presenting with HbA1c > 7.3% (56 mmol/mol) and fasting glucose < 8.33 mmol/l. However, in our study GCK and HNF1A-MODY patients showed comparable levels of fasting glucose, and none of them had a level of fasting glucose > 8.33 mmol/l, except for the HNF1A-MODY patient who presented DKA at diabetes onset.

It may be thought that dysglycaemia in obese children indicates a case of T2D. However, auxological parameters from the MODY group show a significant number of patients presenting with a condition of overweight or obesity. A study on a cohort of obese and overweight patients with a clinical diagnosis of T2D reported that 4.5% of them were actually affected by monogenic diabetes [40]. Our data confirm that BMI may not only be indicative of T2D but also MODY should be suspected if other anamnestic and clinical conditions coexist.

To date, there are few data [41, 42] on OGTT response in children and adolescents affected by MODY. As expected, we found that patients affected by HNF1A-MODY had a markedly higher response to OGTT in terms of two hours postload blood glucose compared to GCK-MODY patients. Indeed, defects of the *GCK* gene cause a higher glycaemic set point of insulin secretion resulting in mild fasting hyperglycaemia, in contrast to other subtypes which are characterized by impaired insulin secretion and high glycaemic response after meals and during OGTT [4].

The coexistence of GCK-MODY and cystic fibrosis is very rare. To the best of our knowledge, only two other cases of children suffering from both cystic fibrosis and GCK-MODY have been reported in literature [43, 44]. It is well known that patients with cystic fibrosis have a gradual deterioration in their glucose tolerance status that begins in the first years of the disease leading to the onset of cystic fibrosis related diabetes (CFRD) [45]. This type of diabetes is characterized by clinical signs at the onset that are very similar to those of MODY. Our experience highlights the need to consider monogenic diabetes also in children and adolescents with cystic fibrosis. Recognition of non-CFRD forms of diabetes in these patients is crucial in planning the most suitable treatment and follow-up.

Limitations to this study involve the retrospective design and the restriction to the sole population of Southern Italy, mostly composed of Caucasian ethnicity. Unfortunately, some clinical data during the follow-up period, such as C-peptide levels, are not available. Finally, due to the technology used for genetic investigations, some gene variations characterized by large gene deletions may have been overlooked. This group of large genomic rearrangements, including 17q12 microdeletions, have been described in literature as responsible for the majority of cases of HNF1B-MODY, known as MODY 5 [46, 47].

## Conclusions

Our findings suggest that a detailed clinical evaluation (i.e. family history, phenotypic features, diabetes-specific autoantibodies response, and assessment of endogenous insulin secretion) could be more useful than restricted criteria as a first approach to screen diabetic children who need genetic testing for MODY. Finally, we speculate that easier and less expensive approaches to genetic investigations will allow to diagnose an increasing number of cases in the near future. A correct, prompt diagnosis is essential to start the most appropriate treatment, to offer adequate genetic counselling, and also to facilitate the diagnosis of monogenic diabetes in asymptomatic family members.

## Data Availability

All published data are available if necessary.
